# The nutritional content required to design an educational application for infertile women

**DOI:** 10.1186/s12905-023-02156-y

**Published:** 2023-01-17

**Authors:** Mostafa Langarizadeh, Azadeh Nadjarzadeh, Banafshe Maghsoudi, Seyed Ali Fatemi Aghda

**Affiliations:** 1 Department of Health Information Management, School of Health Management and Information Sciences, Iran University of Medical Sciences, Tehran, Iran; 2 Nutrition and Food Security Research Center, Shahid Sadoughi University of Medical Sciences, Yazd, Iran; 3 Department of Nutrition, School of Public Health, Shahid Sadoughi University of Medical Sciences, Yazd, Iran; 4 Research Center for Health Technology Assessment and Medical Informatics, School of Public Health, Shahid Sadoughi University of Medical Sciences, Yazd, Iran

**Keywords:** Infertility, Nutrition Education, Mobile phone Health (mHealth), Needs assessment

## Abstract

**Background:**

Infertility has been considered as a critical health problem for couples and society. Given the worldwide exponential rise of infertility, mobile phone-based applications are drastic ways to deliver nutrition educational content to women with infertility. The present study aimed to prepare the required educational content for designing a mobile phone-based nutrition educational application for women with infertility.

**Methods:**

Educational contents were initially determined based on the literature review and library studies. As a result, a researcher-made questionnaire was designed containing 28 items in six dimensions. To ensure about the questionnaire’s validity, a panel of experts (15 nutritionists and 5 infertility specialists) was asked to review the items, make revisions (if necessary), and confirm the final contents. The questionnaire reliability was also corroborated using the KR-20 coefficient (0.89).

**Results:**

While the amount of consumed calories per meal and between meals were not significantly effective on fertility, factors such as main definitions, disease and treatment instructions, personal activities and habits, and menstruation were deemed necessary by the respondents. Participants also suggested adding sections entailing introductions to different types of diets, users' suggestions and opinions, and of the address and contact information of senior nutrition centers to the questionnaire.

**Conclusions:**

Followed by obtaining the required valid and reliable contents, a mobile phone- based nutrition education application can be designed to improve the patients' knowledge and facilitate their treatment process.

## Background

The desire for childbearing has always been an inherent fascination of women throughout the history so that reproduction has been inspected as one of the most important goals of marriage and family formation. However, about 50 to 80 million people in the world suffer from some types of infertility [1, 2]. According to the World Health Organization (WHO), one in six couples suffers from infertility, compared with one in four in the developing countries. In Iran, this rate is about 13 to 20 percent, which is higher than the global average [3, 4].

According to the WHO, infertility is defined as not being able to conceive after 12 months of unprotected intercourse [5]. Infertility, a threat to stability and strength of marital relationship and family survival, especially in traditional societies such as Iran [7, 8], = not only affects couples' relationship, but also leads to marital problems [6].

Based on the literature [9, 10], a variety of factors may increase the risk of infertility, including: obesity, smoking, recreational drugs (narcotics and anabolic steroids), alcohol consumption, rising age of marriage and childbearing, psychological stress, poor nutrition, and modern lifestyle consisting of reduced activity, better food accessibility, more desire for food, as well as popularity of fast foods and high-calorie foods. Some of these factors are immutable (age and disease), while others are modifiable (lifestyle and nutrition) [11, 12]. Although women have mostly been blamed for infertility, studies have shown that men and women hold an equal share of 40% in infertility while the final 20% is attributed to other unknown factors [13]. Among other effective factors in female infertility, lifestyle and inappropriate eating habits resulting in overweight, and obesity are of great importance. In confirmation of the traditional Iranian medicine, the modern medicine has also made remarkable strides in investigating the role of nutrition in infertility, reproductive system, infections, and diseases [14–18].

The infertility treatment process is relatively long and patients need a variety of information and trainings. Given that education and knowledge (such as lifestyle changes) are long-term processes that cannot be resolved in short educational courses, frequent visits are required that impose costs on patients in addition to the treatment costs [19–21]. As a result, they may not complete the training process and may even give up treatment. In this regard, the use of electronic communication devices has been found to increase significantly due to industrialization, so that the per capita number of mobile phone devices was expected to reach 1.5 per person in 2022 [22–24]. Mobile phones have played a significant role in expansion of Tele-Education; they improved education, especially in the field of health, provided health services, reduced medical costs, and solved many cultural and religious problems in the society. According to many users, mobile applications are more efficient and attractive than other traditional teaching methods such as lecturing and counseling [25, 26].

“Mobile-based nutrition education application for women with infertility” provides the needed nutrition content for the target community (women with infertility problems) easily and cheaply via mobile phones. Due to the high rate of infertility in Iran, the effects of nutrition and lifestyle on infertility, lack of knowledge, as well as increased use of mobile health programs, this study aimed to design a mobile-based nutrition education application followed by determining the educational needs of women with infertility in terms of their dietary habits, culture etc.

## Materials and methods

In order to determine the educational needs regarding dietary intakes and diet in women with infertility, articles published on nutritional considerations in infertility were searched from the Iranian (Magiran, Civilica) and international (Science Direct, PubMed) databases resulting in a total of 30 studies. The need assessment was conducted using a researcher-made questionnaire including demographic information (3 questions; age, gender, and work experience) and educational contents (28 questions).

The educational contents covered six dimensions of: definitions (3 questions), disease and treatment instructions (4 questions), diet and nutrition (6 questions), dietary habits (9 questions), personal activities and habits (3 questions), and menstruation status (3 questions). At the end of each section, an unstructured question was included to obtain the participants' additional comments. Content validity of the questionnaire was confirmed by a panel of experts including two specialists in medical informatics and health information management, one nutritionist, and one specialist in infertility. Reliability of the questionnaire was also corroborated by KR-20 coefficient (0.89).

The target population included women diagnosed with infertility since they can use the present findings in meeting their disease. The research participants consisted of all nutrition and infertility specialists working in Research and Clinical Center for Infertility, Yazd Shahid Sadoughi University of Medical Sciences, which is one of the greatest scientific and well-equipped centers in Iran. Given geographical location and lower costs of this center compared to specialized centers in other provinces, Yazd Reproductive Sciences Institute receives a large number of patients with various cultures, customs, and traditions from all over the country. Followed by performing the purposeful sampling method, 20 participants were selected including nutrition (n = 15) and infertility (n = 5) specialists. However, a total of 15 questionnaires (12 nutritionists and 3 infertility specialists) were investigated since five physicians (3 nutritionists and 2 infertility) did not complete the administered questionnaires.

The inclusion criteria were having at least 5 years of work experience and cooperation with Infertility Center of Yazd Shahid Sadoughi University. In terms of the exclusion criterion, individuals who were reluctant to participate in the study were removed from data analysis.

To observe ethical considerations, all participants were explained about the study goals and procedures. They were also ensured about confidentiality of information and voluntary participation in the research.

Followed by distributing the needs assessment questionnaire among specialists, the collected data were analyzed using SPSS software version 25. Data analysis was performed using descriptive statistics and educational contents approved by at least 60% of the participants were considered essential.

## Results

Based on the demographic information (Table 1), the rate of participation was higher in nutritionists (67%) than that of the infertility specialists. Majority of the participants (86%) had 40 years of age and higher. Nearly 75% of the respondents had more than 10 years of work experience, which indicates their good experience in the field of infertility. Nutrition education needs were specified under six domains: definitions, disease and treatment instructions, diet and nutrition, eating habits, personal activities and habits, as well as menstruation (Table 2). According to the specialists, 26 items (among 28 items of the initial needs assessment questionnaire) were needed for developing the mobile application. All items in the dimensions of definitions, disease and treatment instructions, personal activities and habits, as well as menstruation were determined necessary. In diet and nutrition dimension, most respondents believed that the items that asked about calorie intake in each meal and interval between meals were not effective on infertility, which were removed from the educational contents.

**Table 1:** Frequency distribution of the participants' demographic characteristics

Demographic information	Participants					
	Nutritionist		Infertility specialist		Total	
	N	%	N	%	N	%
*Age*						
40 <	2	13.3	0	0	2	13.3
40–50	5	33.3	3	20	8	53.3
50 >	3	20	2	13.3	5	33.3
*Gender*						
Female	6	40	3	20	9	60
Male	4	26.6	2	13.3	6	40
10 <	3	20	1	6.6	4	26.6
*Work experience*						
10–20	4	26.6	1	6.6	5	33.3
> 20	3	20	3	20	6	40

**Table 2:** Frequency distribution of participants' answers about nutrition educational content

Row	Educational content	Responses			
		Necessary		Unnecessary	
		N	%	N	%
1	*Definitions*				
	Infertility	15	100	0	0
	Dietary pattern	13	86.6	2	13.4
	Life style	12	80	3	20
2	*Disease and treatment instructions*				
	Hormonal diseases	11	73.3	4	26.7
	Chronic diseases	15	100	0	0
	Consumption of herbal tea and drinks	13	86.6	2	13.4
	Taking medication	15	100	0	0
3	*Diet and nutrition*				
	Importance of nutrition	12	80	3	20
	Effects of obesity	14	93.3	1	6.7
	Special treatment regimen	9	60	6	40
	The amount of calories consumed per meal	6	40	9	60
	Taking supplements and vitamins	10	66.6	5	33.4
	Allergy to certain foods	12	80	3	20
4	*Dietary habits*				
	Consuming fast foods	9	60	6	40
	Consuming fried foods	9	60	6	40
	Consuming fruits and vegetables	12	80	3	20
	Consuming simple carbohydrates	13	86.6	2	13.4
	Consuming of fats	11	73.3	4	26.7
	The interval between meals	7	46.6	8	53.4
	Number of meals per day	10	66.6	5	33.4
	Drinking carbonated beverages	12	80	3	20
	Drinking alcohol	8	53.3	7	46.7
5	*Personal activities and habits*				
	Having physical activity	10	66.6	5	33.4
	Tobacco use (cigarettes and hookahs)	13	86.6	2	13.4
	Using mobile phones	12	80	3	20
6	*Menstruation status*				
	Menstrual status	15	100	0	0
	Menstrual duration	15	100	0	0
	Menstrual severity	15	100	0	0

After collecting, summarizing, and modifying the additional suggestions provided by some respondents about the other required nutritional information, the participants were asked to review and confirm them. Unfortunately, of 15 specialists, only 10 individuals responded to these suggestions. Table 3 represents the additional suggestions provided by some respondents. At this stage, only 'introduction of infertility clinics and centers in Iran' did not obtain the required score as a necessary item (60%), but the rest of the items were determined as necessary.

**Table 3:** Frequency distribution of participants' responses to suggestions

Row	Suggestions	Responses			
		Necessary		Unnecessary	
		N	%	N	%
1	Introducing different types of diets	7	70	3	30
2	Introducing infertility clinics and centers in Iran	4	40	6	60
3	adding users' suggestions and comments section	7	70	3	30
4	Introducing senior nutrition centers for nutritional advice	8	80	2	20

## Discussion

Most related studies in the literature scrutinized the effect of nutrition on infertility, the importance of educating infertile couples, the creation of a minimum data set for patients with infertility, the development of electronic health record in infertility with a traditional medicine approach, etc. In other words, no study has ever investigated designing a nutrition education application to the best of our knowledge in Iran [17, 27, 28].

Given that patients need comprehensive information about their disease nature, complications, and treatment process, education can be considered an effective tool in controlling and improving the disease, reducing costs, and raising public knowledge. Based on the studies conducted in different parts of the world [29, 30], couples with infertility do not have enough knowledge about their disease, which has created a great obstacle in the treatment process.

Lemoine et al. [28] examined the information needs of fertile people in Canada and noted that people seeking fertility and infertility services received a variety of diagnostic and therapeutic information and people whose information needs were met achieved better psychological outcomes. As Nowruz et al. [29] reported, approximately half of patients had limited knowledge about infertility-related factors. They concluded that designing, implementing, and evaluating an appropriate training program is necessary to improve patients' knowledge about infertility. The present study findings not only support results achieved by previous studies but also determine the significantly needed educational contents using the panel of experts in this field.

Mostajeran et al. [27] investigated symptoms of infertility and its predisposing factors from the viewpoint of traditional Iranian medicine. Upon their findings, applying new prevention and treatment strategies, modifying lifestyle and eating habits as well as using herbal medicines can be effective in treating infertility. According to the study conducted by Sadeghi et al. [31], using simple carbohydrates and sweets was higher in infertile women while fiber intake was significantly higher in healthy women. Furthermore, overweight and obesity was 2.2 times more prevalent among infertile women compared with the healthy women and a significant difference was observed between women with and without infertility in terms of mean weight and body mass index (BMI). Similarly, intake of herbal medicines and traditional Iranian medications was esteemed necessary by our respondents.In addition to considering these cases, the present study has considered educational dimensions more comprehensively.

A study [15] over the relationship of individuals' dietary factors and preconceptions with fertility indicated that consuming more fruits along with reducing the intake of fast food and sugary drinks improved pregnancy. This suggests the significant role of foods and dietary patterns in fertility, which has recently attracted more attention in nutrition education. In a study by Jahangirifard et al. [32], the relationship between dietary patterns and fertility outcomes was examined among infertile women. The results showed that diet adherence was higher in fertile women. They also stated that nutritional interventions before attempting to treat infertility improved the treatment outcomes. In the same vein, Alizadeh et al. [33] concluded that the rate of fertility was six times higher in people with appropriate diet and lifestyle, so that consumption of unsaturated fatty acids, fruits, vegetables, and low-fat dairy products improved fertility. These studies provide a robust theoretical background in support of the educational contents recommended by participants of the present study in the field of diet and eating habits.

Given the exponential growth of new technologies, such as mobile phone applications in various fields, designing and implementing mobile phone-based training programs is an efficient way to raise knowledge and conduct training programs. The mobile health has found a great place in the field of health education and treatment for different diseases.

In a similar study [34] that targeted at developing educational contents for patients with epilepsy, specialists and patients confirmed the necessity of educational content in three areas of disease information, lifestyle, and medications.

The relationship between using mobile-phone applications and reproductive knowledge among Australian women was examined by Ford et al. [29]. As they stated, women with unsuccessful conception or infertility problems sought related information, observed treatment considerations, attended training courses, and tried to improve their knowledge more frequently than the healthy women. Preliminary evidences corroborate usefulness of mobile-phone as a medium to provide fertility information to women. Oostingh et al. [35] designed the coaching program of Smarter Pregnancy on the Android platform and studied the impact of nutrition education on infertile couples undergoing in vitro fertilization. They found that inappropriate lifestyle and nutrition behaviors improved among users of this application so that their poor nutrition habits and fruit consumption improved by 73% and 55%, respectively. Findings of the above-mentioned studies were in the same line with the present study.

Similar to the present study design, Sheikhtaheri et al. (36) conducted an educational needs analysis for parents of children with acute lymphoblastic leukemia. However, we investigated women with infertility and paid special attention to the field of nutrition-based educational content.

The present study aimed at providing the necessary educational content according to the patients’ needs and eating culture and habits of Iranian population.

Our study was subject to some limitations. Some experts did not cooperate in completing the questionnaire due to lack of time and knowledge about the study objectives. We tried to meet this problem by providing them with an introduction letter from Yazd University of Medical Science, including the study objectives and procedure. Considering the current prevalence of Corona Virus Disease pandemic throughout the world, data collection was only limited to Yazd Infertility Center.

## Conclusion

One of the most important initial steps in designing an educational application is to determine and develop to the point contents based on the educational needs of the target population. In the present study, educational contents of a mobile phone-based nutrition application for women with infertility problems were specified and confirmed by reviewing the related literature and collecting opinions of a panel of nutrition and infertility specialists in Yazd Infertility Center, respectively. Findings of this study can be beneficial for the health care institutions and providers to design an effective application.

## Data Availability

The data used and analyzed during the current study are not publicly available due Shahid Sadoughi University of Medical Sciences policy but are available from the corresponding author on reasonable request.
